# SphK-produced S1P in somatic cells is indispensable for LH-EGFR signaling-induced mouse oocyte maturation

**DOI:** 10.1038/s41419-022-05415-2

**Published:** 2022-11-17

**Authors:** Feifei Yuan, Xiaoqiong Hao, Yanying Cui, FuXin Huang, Xiaodan Zhang, Yanli Sun, Tiantian Hao, Zhijuan Wang, Wei Xia, Youqiang Su, Meijia Zhang

**Affiliations:** 1 Division of Cell, Developmental and Integrative Biology, School of Medicine, South China University of Technology, Guangzhou, 510006 P.R. China; 2 Department of Physiology, Baotou Medical College, Baotou, 014000 P.R. China; 3 Department of Reproductive Medicine Centre, Guangzhou First People’s Hospital, South China University of Technology, Guangzhou, 510180 P.R. China; 4 Shandong Provincial Key Laboratory of Animal Cells and Developmental Biology, School of Life Sciences, Shandong University, Qingdao, 266237 P.R. China

**Keywords:** Infertility, Reproductive disorders

## Abstract

Germ cell division and differentiation require intimate contact and interaction with the surrounding somatic cells. Luteinizing hormone (LH) triggers epidermal growth factor (EGF)-like growth factors to promote oocyte maturation and developmental competence by activating EGF receptor (EGFR) in somatic cells. Here, we showed that LH-EGFR signaling-activated sphingosine kinases (SphK) in somatic cells. The activation of EGFR by EGF increased S1P and calcium levels in cumulus-oocyte complexes (COCs), and decreased the binding affinity of natriuretic peptide receptor 2 (NPR2) for natriuretic peptide type C (NPPC) to release the cGMP-mediated meiotic arrest. These functions of EGF were blocked by the SphK inhibitor SKI-II, which could be reversed by the addition of S1P. S1P also activated the Akt/mTOR cascade reaction in oocytes and promoted targeting protein for Xklp2 (TPX2) accumulation and oocyte developmental competence. Specifically depleting *Sphk1/2* in somatic cells reduced S1P levels and impaired oocyte meiotic maturation and developmental competence, resulting in complete female infertility. Collectively, SphK-produced S1P in somatic cells serves as a functional transmitter of LH-EGFR signaling from somatic cells to oocytes: acting on somatic cells to induce oocyte meiotic maturation, and acting on oocytes to improve oocyte developmental competence.

## Introduction

Oocytes enter meiosis during fetal life and are long-term arrested in prophase I. When the follicles develop into the antral stage, oocytes become competent to resume meiosis. Natriuretic peptide type C (NPPC, also known as CNP) from mural granulosa cells (MGCs) maintains oocyte meiotic arrest by binding to its cognate receptor natriuretic peptide receptor 2 (NPR2) to stimulate cGMP production [1–4]. In preovulatory follicles, luteinizing hormone (LH) activates cGMP hydrolysis [5] and decreases guanylyl cyclase activity [6], resulting in a cGMP decrease in the follicles and then meiotic resumption. LH also decreases *Nppc* mRNA levels by activating tristetraprolin, which would lead to a decrease in NPPC levels and participate in meiotic resumption. LH signaling is amplified by promoting the expression of epidermal growth factor (EGF)-like growth factors in mural granulosa cells (MGCs) to activate EGF receptor (EGFR) in cumulus cells [7, 8], which is essential for oocyte maturation, cumulus expansion, and ovulation. The activation of EGFR by EGF increases calcium levels in cumulus cells to decrease the binding affinity of NPR2 for NPPC, resulting in meiotic resumption [9, 10]. In addition, elevated calcium also promotes cumulus expansion.

Oocyte maturation, completion of fertilization, and the first stages of embryo cleavage almost completely depend on the translation of maternal mRNAs [11], which is controlled by the regulators from the oocyte itself and somatic cells [12]. LH-EGFR signaling in somatic cells activates the PI3K/Akt/mTOR pathway in oocytes [12], and then stimulates the translation of eIF4E-sensitive mRNAs characterized by a 5’ terminal oligopyrimidine (TOP) motif [13]. The synthesis of these special proteins is required for normal spindle morphology and chromosome alignment during oocyte maturation [13], which is involved in establishing oocyte competence to successfully develop into embryos [12]. However, somatic cell-dependent maternal mRNA translation in oocytes is dispensable for meiotic progression [13]. Targeting protein for Xklp2 (TPX2) is a key component in spindle assembly, whose translation depends on somatic cell inputs and is promoted by the RNA-binding protein deleted in azoospermia-like (DAZL) [12, 14]. The downregulation of TPX2 or DAZL in germinal vesicle (GV) oocytes causes defects in spindle assembly and embryonic cleavage [14, 15].

Sphingosine-1-phosphate (S1P), a bioactive sphingolipid, is tightly regulated in a spatial-temporal manner by the synthetase sphingosine kinases (SphK1 and SphK2) and the degrading enzymes sphingosine phosphate lyase 1 (SGPL1) and S1P phosphatases (SGPP1 and SGPP2) [16]. The levels of cellular S1P are low in the basal state and are increased rapidly and transiently when cells are exposed to growth factors, including EGF [17]. S1P can mobilize endoplasmic reticulum (ER) calcium storage to elevate calcium levels by activating specific high-affinity G-protein-coupled receptors (S1PRs) or by acting intracellularly [18], which is of crucial importance in regulating cell function, including the reduced NPR2 activity in cumulus cells for oocyte meiotic resumption [9, 10, 19]. S1P can also activate the PI3K/Akt/mTOR pathway in various cell types via S1PRs, and then regulate cell growth by affecting the crucial steps of protein synthesis [20–22].

LH-EGFR signaling in somatic cells plays an important role in oocyte meiotic maturation and developmental competence. The precise molecular targets in these processes remain unclear. Here, we demonstrate that LH-EGFR signaling activates SphK to increase S1P levels in somatic cells. S1P not only induces oocyte meiotic maturation by elevating calcium levels of somatic cells, but also promotes oocyte developmental competence, possibly by activating the Akt/mTOR pathway in oocytes.

## Results

### LH/hCG and EGF increase SphK activity and S1P levels

**Fig. 1:**
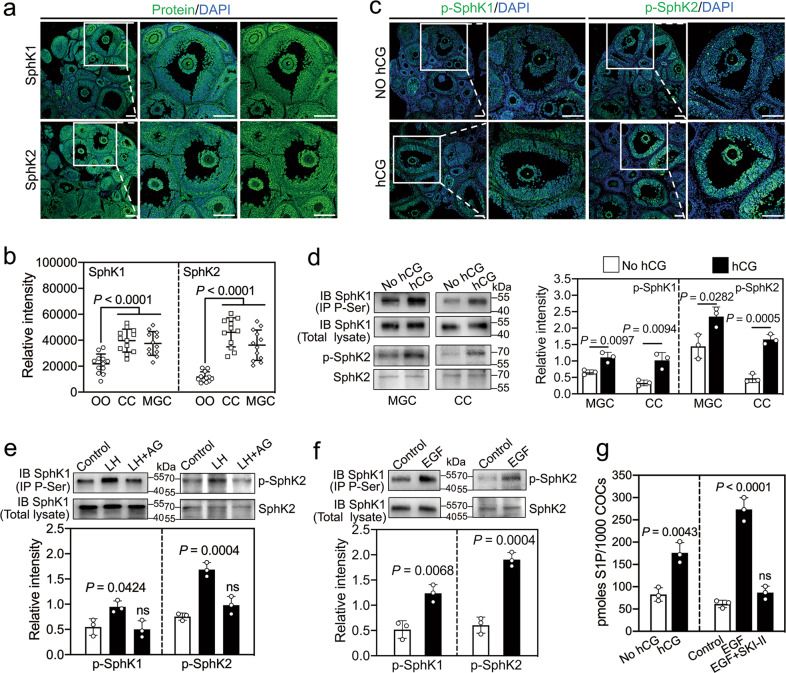
LH/hCG and EGF increases phosphorylated SphK and S1P levels. **a** Representative images of SphK1 and SphK2 (green) staining in ovaries isolated from eCG-primed mice. The nuclei were counterstained with DAPI (blue). The enlarged views of the boxed area are shown on the right side. **b** Fluorescence intensity analysis of SphK1 and SphK2 in oocytes (OO), cumulus cells (CC), and MGCs. A total of 12 large antral follicles were scored. **c** Representative images of p-SphK1 and p-SphK2 (green) staining in ovaries isolated from the mice before (no hCG group) and after hCG treatment (2 h). The nuclei were counterstained with DAPI (blue). The enlarged views of the boxed area are shown on the right side. **d–f** Western blotting analysis of p-SphK1 and p-SphK2 levels in MGCs and/or cumulus cells isolated from the mice before and after hCG treatment (2 h, **d**), the follicles at 2 h of culture (**e**) and the COCs at 2 h of culture (**f**). For p-SphK1, cell lysates were immunoprecipitated with anti-P-Ser antibodies and immunoblotted for SphK1. SphK1 in total lysates was used as an internal control. For p-SphK2, total SphK2 was used as an internal control. **g** The effects of hCG and EGF on the S1P levels of COCs. The concentration of SKI-II was 20 μM. For **a–g**, *n* = 3 independent experiments. The data represent the mean ± s.d. *P* values were determined by two-sided Student’s *t*-test (**d**, **f**, left panel of **g**) and one-way ANOVA followed by Tukey’s test (**b**, **e**, right panel of **g**). ns, not significant. Scale bars, 100 μm.

We first detected the localization of SphK in eCG-primed mouse ovaries by immunofluorescence. SphK1/2 were strongly expressed in the cytoplasm of MGCs, cumulus cells, theca cells, and stromal cells and were also expressed in the cytoplasm and nucleolus of oocytes (Figs. 1a and S1a). The analysis of fluorescence intensity indicated that the expression levels of SphK1/2 in MGCs and cumulus cells were significantly higher than those in oocytes (Fig. 1b). The activation of the LH receptor by human chorionic gonadotropin (hCG, a pregnancy hormone that exhibits LH activity) obviously increased the fluorescence intensity of phosphorylated SphK1/2 in MGCs and cumulus cells (Figs. 1c and S1a). Consistent with this finding, the protein levels of phosphorylated SphK1/2 were significantly increased in MGCs and cumulus cells after hCG treatment (Fig. 1d). Furthermore, the addition of LH to follicle culture and EGF to cumulus-oocyte complex (COC) culture significantly increased the protein levels of phosphorylated SphK1/2 in cumulus cells (Fig. 1e, f). The LH-induced increase in phosphorylated SphK1/2 was completely blocked by the EGF receptor inhibitor AG1478 (Fig. 1e), indicating that LH activates SphK via EGFR. Treatment with hCG significantly increased S1P levels in COCs (Fig. 1g). EGF also significantly increased S1P levels in cultured COCs, which was completely inhibited by the SphK inhibitor SKI-II (Fig. 1g). Therefore, LH/hCG activates SphK in granulosa cells via EGFR signaling, which could increase intracellular S1P levels.

### SphK activity is required for LH-EGFR signaling-induced oocyte meiotic resumption and cumulus expansion

**Fig. 2:**
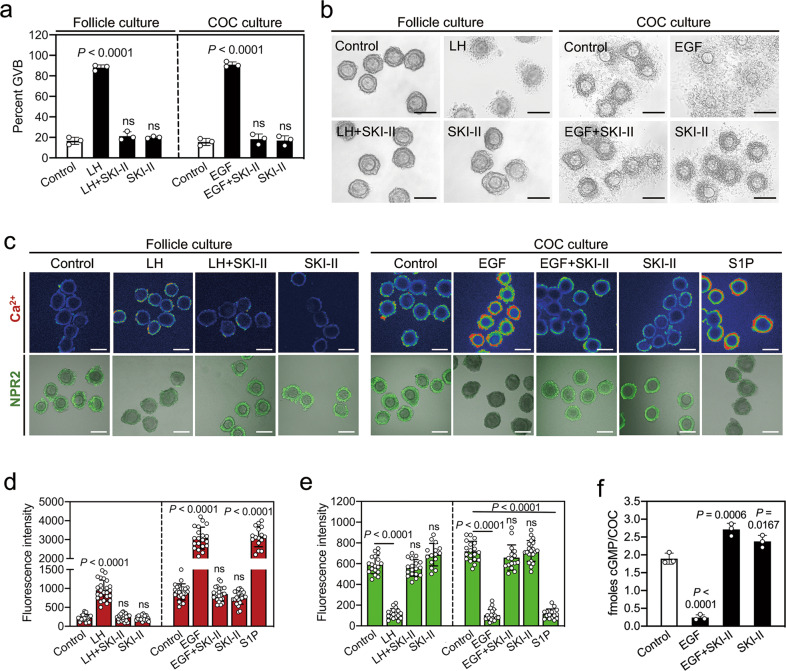
SKI-II blocks LH/EGF-induced oocyte meiotic resumption and cumulus expansion. Follicles and COCs isolated from eCG-primed mice were cultured in a medium supplemented with different drugs. **a** Proportion of oocytes having undergone GVB was determined at 4 h of culture. (*n* = 3 independent experiments). **b** Representative images of the morphology of cumulus expansion at 8 h of follicle culture and at 15 h of COC culture. At the end of the follicle culture, the COCs were released from the follicles. (*n* = 3 independent experiments). **c–e** Representative images of calcium levels (pseudocolor) and NPR2 affinity for NPPC (green) in cumulus cells at 2 h of culture (**c**), and the statistical analysis of calcium levels (**d**), and NPR2 affinity (**e**). (*n* ≥ 15 independent samples). **f** The cGMP levels of COCs after different treatments. (*n* = 3 independent experiments). The data represent the mean ± s.d. *P* values were determined by one-way ANOVA followed by Tukey’s test (**a**, **d**–**f**). ns not significant. Scale bars, 100 μm.

We studied the effect of SphK activity on LH-EGFR signaling-induced oocyte meiotic maturation. Interestingly, SKI-II, inhibited LH- and EGF-induced oocyte meiotic resumption in a dose-dependent manner (Fig. S1b), and 20 μM SKI-II completely blocked LH- and EGF-induced oocyte meiotic resumption, cumulus expansion, and cumulus expansion-related gene expression (Figs. 2a, b and S1c, d). However, ryanodine receptor inhibitor tetracaine had no effect, and the IP3 receptor inhibitors 2-aminoethoxydiphenyl borate (2-APB) and heparin had a slight inhibitory effect on EGF-induced meiotic resumption (Fig. S1e). SKI-II also completely blocked the effects of LH and/or EGF on calcium levels, NPR2 affinity for NPPC, and cGMP levels in COCs (Fig. 2c–f). The addition of S1P reversed the inhibition of SKI-II on EGF functions (Fig. S2). The elevation of calcium levels can induce oocyte meiotic resumption and cumulus expansion [9, 10]. These results suggest that LH-EGFR signaling induces oocyte meiotic resumption and cumulus expansion by SphK-producing S1P.

### LH-EGFR signaling increases PLN protein levels via SphK-produced S1P

**Fig. 3:**
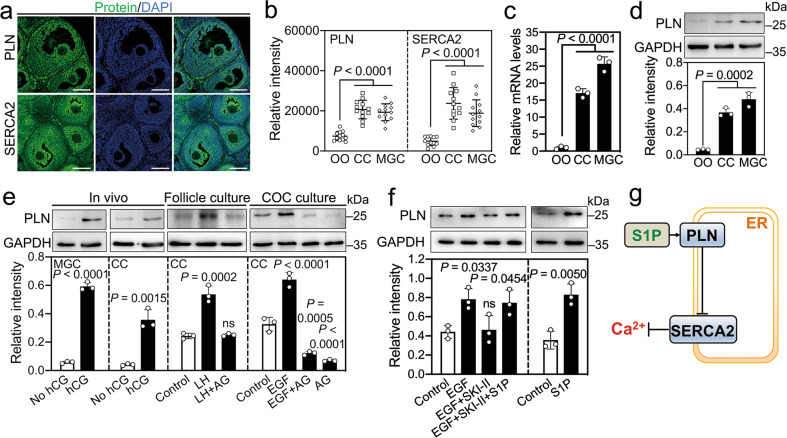
LH-EGFR signaling and S1P increase the protein levels of PLN. **a** Representative images of PLN and SERCA2 (green) staining in the ovaries isolated from eCG-primed mice. The nuclei were counterstained with DAPI (blue). Scale bars, 100 μm. **b** Fluorescence intensity analysis of PLN and SERCA2 in oocytes (OO), cumulus cells (CC), and MGCs. A total of 12 large antral follicles were scored. **c**, **d** The mRNA (**b**) and protein (**c**) levels of PLN in oocytes, cumulus cells, and MGCs isolated from eCG-primed mice. **e** Western blotting analysis of PLN levels in MGCs and/or cumulus cells isolated from the mice before and after hCG treatment (2 h), the follicles at 2 h of culture, and the COCs at 2 h of culture. **f** Western blotting analysis of PLN levels in cumulus cells of COCs at 2 h (right panel) or 0.5 h (left panel) of culture. GAPDH was used as an internal control (**d–f**). **g** Model of the signaling pathway for S1P elevating intracellular calcium levels. The endoplasmic reticulum (ER) is represented as an orange square. S1P increases PLN levels to inhibit SERCA2 activity, thereby increasing the intracellular Ca^2+^ concentration. For **a–f**, *n* = 3 independent experiments. The data represent the mean ± s.d. *P* values were determined by two-sided Student’s *t*-test (left two panels of **e**, right panel of **f**) and one-way ANOVA followed by Tukey’s test (**b–d**, right two panels of **e**, left panel of **f**). ns not significant.

Phospholamban (PLN), sarcolipin (SLN), and myoregulin (MLN) are reversible endogenous inhibitors of sarcoplasmic/endoplasmic reticulum calcium ATPase 2 (SERCA2) [23], and the inhibition of SERCA2 activity could increase the calcium levels in cumulus cells [9, 10]. Thus, we detected the expression patterns of PLN, SLN, and MLN in the ovary. *Pln* mRNA was present at a higher concentration than mRNAs for *Sln* and *Mln* in granulosa cells (Fig. S3a). The immunofluorescence results showed that PLN and SERCA2 were strongly colocalized in the cytoplasm of MGCs and cumulus cells, theca cells, and stromal cells but were slightly expressed in oocytes (Fig. 3a, b). In line with these observations, the mRNA and protein levels of PLN were significantly higher in MGCs and cumulus cells than in oocytes (Fig. 3c, d). Treatment with hCG had no effect on *Pln* mRNA levels, but significantly increased the protein levels of PLN in MGCs and cumulus cells (Figs. 3e and S3b, c). Similarly, the addition of LH to follicle culture and EGF to COC culture significantly increased the protein levels of PLN in cumulus cells, which was completely reversed by AG1478 (Fig. 3e). Furthermore, SKI-II completely inhibited EGF-promoted PLN protein levels, which could be reversed by the addition of S1P (Figs. 3f and S3d). Collectively, LH-EGFR signaling-produced S1P increases PLN levels in cumulus cells, and then elevates intracellular calcium levels by inhibiting SERCA2 activity (Fig. 3g). *S1pr1* and *S1pr2* mRNAs were present at high concentrations in MGCs and cumulus cells, and *S1pr3* mRNA was present at high concentrations in oocytes (Fig. S4a). However, the inhibition of S1PR1 (by W146) and/or S1PR2 (by JTE-013) could not block EGF- and S1P-induced increases in PLN and calcium levels in cumulus cells (Fig. S4b–d).

### *Sphk1/2* depletion in granulosa cells causes oocyte meiotic maturation defects and infertility

**Fig. 4:**
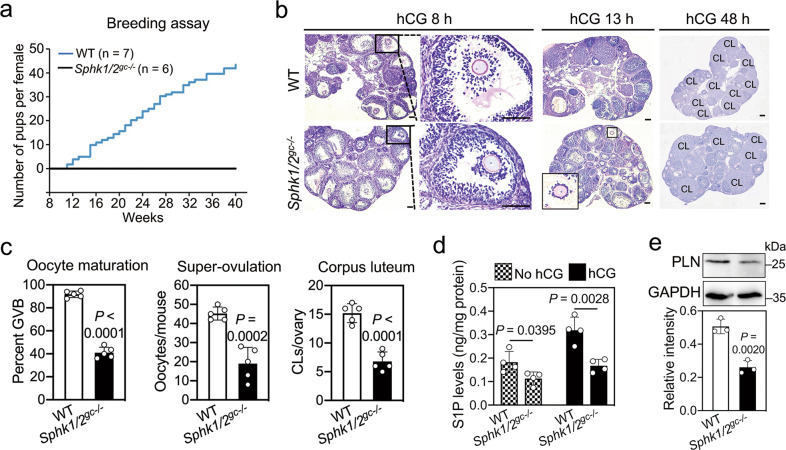
*Sphk1/2* depletion in granulosa cells impairs oocyte meiotic maturation and female fertility. **a** Cumulative numbers of pups born per female among the WT (*n* = 7) and *Sphk1/2*^*gc−/−*^ (*n* = 6) females during the 8-month period of a fertility test. **b** PAS staining showing histological changes during hCG-induced ovulation and luteinization in WT and *Sphk1/2*^*gc−/−*^ ovaries. Representative images are shown. The enlarged views of the black boxed area are shown on the right side (hCG 8 h group), and the enlarged view of the black boxed area is shown in the lower left corner (hCG 13 h group). CL corpus luteum. Scale bars, 100 μm. **c** Proportions of oocytes having undergone GVB at 8 h post-hCG, ovulated oocytes at 13 h post-hCG, and corpus luteum at 48 h post-hCG in WT and *Sphk1/2*^*gc−/−*^ mice. (*n* = 5 independent experiments). **d** The levels of S1P in ovaries isolated from WT and *Sphk1/2*^*gc−/−*^ mice before and after hCG treatment (2 h). (*n* = 4 independent experiments). **e** Western blotting analysis of PLN levels in cumulus cells isolated from WT and *Sphk1/2*^*gc−/−*^ mice at 2 h post-hCG. GAPDH was used as an internal control. (*n* = 3 independent experiments). The data represent the mean ± s.d. *P* values were determined by two-sided Student’s *t*-test (**c**–**e**).

*Sphk1*- and *Sphk2*-null mice are viable and fertile, but double knockout mice are embryonic lethal [24]. EGF could induce meiotic resumption of oocytes collected from *Sphk1*^*−/−*^ or *Sphk2*^*−/−*^ mice (Fig. S5a), indicating that both SphK1 and SphK2 are indispensable for mouse oocyte maturation. To analyze the physiological function of SphK in the ovaries, we crossed *Sphk2* ^*fl/fl*^ mice with *Fshr-Cre* transgenic mice and then crossed them into the *Sphk1*^*−/−*^ background, yielding *Sphk1*^*−/−*^*; Sphk2* ^*fl/fl*^*; Fshr-Cre* (*Sphk1/2*^*gc−/−*^) mice (Fig. S5b, c). The efficient depletion of *Sphk2* in granulosa cells was confirmed by immunofluorescence and western blotting (Fig. S5d, e). *Sphk1/2*^*gc−/−*^ females were completely infertile (Fig. 4a). Quantification of the number of large antral follicles from eCG-primed mice indicated that the depletion of *Sphk1/2* in granulosa cells had no effect on follicular growth (Fig. S6a, b). Compared with WT mice, the mRNA levels of cumulus expansion-related genes (Fig. S6e), the rate of oocytes having undergone germinal vesicle breakdown (GVB) in preovulatory follicles (91.8 vs. 41.0%, Fig. 4b, c), and the number of ovulated oocytes (45.2 vs. 19.0, Fig. 4b, c) were significantly decreased in *Sphk1/2*^*gc−/−*^ mice after hCG treatment. Consistent with these findings, the number of corpora lutea (CLs) in *Sphk1/2*^*gc−/−*^ mice was significantly decreased in pubertal ovaries at 48 h post-hCG and in adult ovaries (Figs. 4b, c and S6c, d). The serum progesterone (P4) levels of *Sphk1/2*^*gc−/−*^ mice were also significantly lower than those of WT mice (Fig. S6f), possibly due to the reduced CLs. Next, we studied the effects of *Sphk1/2* depletion on S1P and PLN levels. In WT mice, hCG treatment for 2 h increased S1P levels in the ovaries and PLN protein (but not mRNA) levels in granulosa cells, but these increases were absent in *Sphk1/2*^*gc−/−*^ mice (Figs. 4d, e and S6g). Consistent with these findings, the intracellular calcium levels were significantly lower and the binding affinity of NPR2 for NPPC was significantly higher in cumulus cells of *Sphk1/2*^*gc−/−*^ mice than those of WT mice (Fig. S7a–c). However, the mRNA and protein levels of NPPC and NPR2 were not obviously different between *Sphk1/2*^*gc−/−*^ and WT mice at 4 h post-hCG (Fig. S7d–f). Thus, the low levels of S1P in *Sphk1/2*^*gc−/−*^ mice could not increase PLN and calcium levels in cumulus cells, resulting in defects in oocyte meiosis maturation and ovulation.

**Fig. 5:**
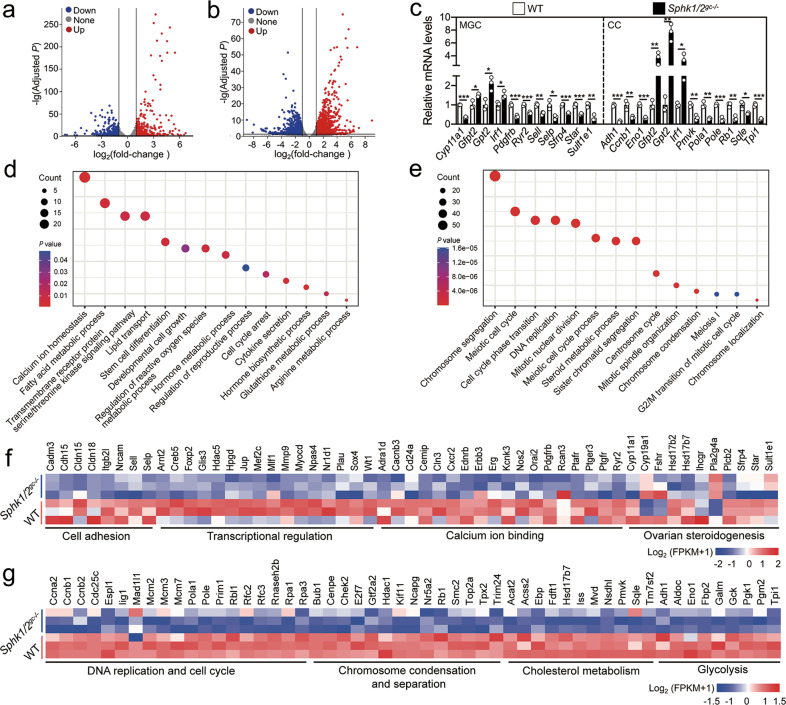
*Sphk1/2* depletion in granulosa cells impairs the integrity of transcriptome. **a**, **b** Volcano plot illustrating the differentially expressed transcripts in MGCs (**a**) and cumulus cells (CC, **b**) isolated from WT and *Sphk1/2*^*gc−/−*^ mice at 2 h post-hCG. **c** Quantitative RT-PCR validating changes in the representative transcripts selected from RNA-seq data. (*n* = 3 independent experiments). The data represent the mean ± s.d. *P* values were determined by a two-sided Student’s *t*-test. (*P < 0.05, **P < 0.01, and ***P < 0.001). **d**, **e** Bubble chart illustrating the enriched GO terms associated with the significantly downregulated transcripts in *Sphk1/2*^*gc−/−*^ MGCs (**d**) and cumulus cells (**e**) identified by RNA-seq. Transcripts with a fold-change ≥2 and a *P* value ≤0.05 were selected for analysis. **f**, **g** Heatmaps illustrating differences between WT and *Sphk1/2*^*gc−/−*^ MGCs (**f**) and cumulus cells (**g**) in the expression of a group of transcripts involved in various processes.

To further identify the mechanism of SphK1/2 on oocyte maturation, transcriptomic analysis was performed on the MGCs and cumulus cells of *Sphk1/2*^*gc−/−*^ and WT mice at 2 h post-hCG. A total of 874 transcripts (including 390 upregulated and 484 downregulated transcripts) in MGCs and 2113 transcripts (including 1195 upregulated and 918 downregulated transcripts) in cumulus cells were differentially expressed in *Sphk1/2*^*gc−/−*^ mice compared with WT mice (Fig. 5a, b and Tables S1, 2). These changes in the expression of representative transcripts were validated by qRT–PCR (Fig. 5c). Gene enrichment analysis revealed that the downregulated transcripts in *Sphk1/2*^*gc−/−*^ MGCs mainly controlled cell adhesion, transcriptional regulation, calcium ion binding, and ovarian steroidogenesis (Figs. 5d, f and S8a, c), which differs from those enriched by the upregulated transcripts (Fig. S8g). Downregulation of these transcripts in the key processes could impair the proliferation, differentiation, and survival of MGCs [25]. In particular, the downregulation of *Sult1e1*, *Cyp11a1*, *Sfrp4*, and *Star* could lead to defects in luteal cell formation (Fig. 5f). The downregulated transcripts in *Sphk1/2*^*gc−/−*^ cumulus cells mainly controlled DNA replication and cell cycle, chromosomal condensation and separation, cholesterol metabolism, and glycolysis (Figs. 5e, g and S8b, d–f), which differs from those enriched by the upregulated transcripts (Fig. S8h). Changes in the expression of the transcripts in these key processes, particularly the downregulation of *Pmvk*, *Sqle*, *Eno1*, and *Tpi1*, could impair the growth [26] and metabolic progress [27] of cumulus cells (Fig. 5g). These findings suggest that the functional impairments of MGCs and cumulus cells in *Sphk1/2*^*gc−/−*^ mice may also contribute to defects in both oocyte meiotic maturation and developmental competence.

### *Sphk1/2* depletion in granulosa cells impairs oocyte developmental competence

**Fig. 6:**
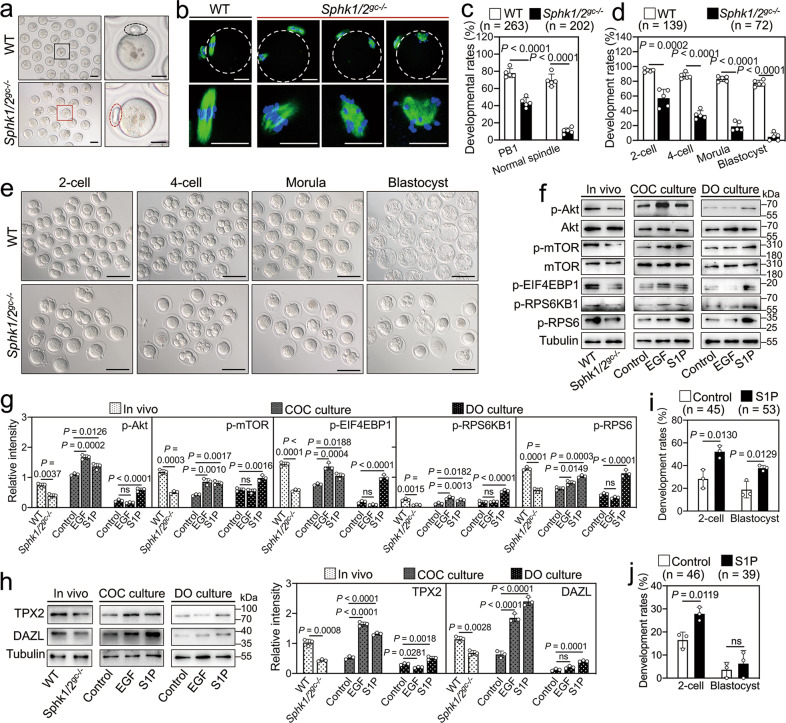
*Sphk1/2* depletion in granulosa cells impairs oocyte developmental competence. **a** Representative images of oocytes collected from the oviducts of WT and *Sphk1/2*^*gc−/−*^ mice at 13 h post-hCG. The enlarged views of the black and red boxed area are shown on the right side. The black dotted circle represents normal PB1, and the red dotted circle represents abnormal PB1 (large PB1). **b** Immunofluorescent staining of α-tubulin showing spindle assembly in MII oocytes ovulated by WT and *Sphk1/2*^*gc−/−*^ mice. The enlarged views indicate the morphology of microtubules (green) and chromosomes (blue). Representative images are shown. **c** Quantification of the rates of PB1 emission and normal spindle formation in oocytes ovulated by WT and *Sphk1/2*^*gc−/−*^ mice. The numbers of analyzed oocytes are indicated (*n*). **d**, **e** Developmental rates (**d**) and representative images (**e**) of preimplantation embryos from WT and *Sphk1/2*^*gc−/−*^ mice. The numbers of analyzed embryos are indicated (*n*). **f**, **g** Western blotting analysis of the levels of p-Akt, p-mTOR, and mTOR downstream effectors p-EIF4EBP1, p-RPS6KB1, and p-RPS6 in oocytes from different treatments. Oocytes were collected from the ovaries at 3 h post-hCG, the COCs at 2.5 h of culture, and the DOs at 1.5 h of culture. Total Akt and mTOR were used as the corresponding internal controls for p-Akt and p-mTOR, and tubulin was used as an internal control for p-RPS6KB1, p-RPS6, and p-EIF4EBP1. **h** Western blotting analysis of the levels of TPX2 and DAZL in MII oocytes from different treatments. MII oocytes were collected from the ovaries at 13 h post-hCG, the COCs at 16 h of culture, and the DOs at 16 h of culture. For the S1P treatment group, COCs or DOs isolated from eCG-primed mice were cultured in a medium with S1P for 0.5 h and then cultured in an S1P-free medium for an additional 15.5 h. Tubulin was used as an internal control. **i**, **j** Quantification of the rates of two-cell stage embryos and blastocyst stage embryos in WT (**i**) and *Sphk1/2*^*gc−/−*^ (**j**) mice. COCs collected from eCG-primed mice were matured in vitro with or without S1P, and then were fertilized in vitro. The numbers of analyzed embryos are indicated (*n*). The data represent the mean ± s.d. *P* values were determined by two-sided Student’s *t*-test (**c**, **d**, **g–j**) and one-way ANOVA followed by Tukey’s test (**g**, **h**). ns not significant.

*Sphk1/2*^*gc−/−*^ females could ovulate but were completely infertile. Thus, we collected oocytes from the oviducts and analyzed their potential defects. Compared with WT mice, the rate of PB1 emission was significantly decreased in *Sphk1/2*^*gc−/−*^ mice (77.9 vs. 43.7%, Fig. 6a, c). Oocytes ovulated by *Sphk1/2*^*gc−/−*^ mice did not complete the first meiosis normally: 89.3% of oocytes either formed abnormal metaphase II (MII) spindles (Fig. 6b) or did not complete cytokinesis and remained at telophase I (Fig. S9a, b). These defects were recapitulated when the oocytes of *Sphk1/2*^*gc−/−*^ mice underwent maturation in vitro (Fig. S10). Interestingly, 33.3% of MII oocytes in *Sphk1/2*^*gc−/−*^ mice exhibited rod-shaped polar body-1 (large PB1) (Fig. 6a), which may be related to poor oocyte quality [28]. Next, we detected oocyte developmental competence. Oocytes ovulated by *Sphk1/2*^*gc−/−*^ mice could be fertilized, but the rate of two-cell embryos was significantly decreased compared with that in WT mice (57.2 vs. 94.8%, Fig. 6d, e). Further development of the two-cell stage embryos into blastocysts was severely impaired in *Sphk1/2*^*gc−/−*^ mice, with the rate of blastocyst formation was only 4.8%, which was significantly lower than the 77.4% in WT mice (Fig. 6d, e). DAPI staining demonstrated that the number of total cells in the blastocysts from *Sphk1/2*^*gc−/−*^ mice was significantly lower than that in the blastocysts from WT mice (42.2 vs. 87.8, Fig. S9c, d).

LH-EGFR signaling can improve oocyte developmental competence by activating the Akt/mTOR pathway in oocytes [12, 13]. Compared with WT mice, the protein levels of p-Akt, p-mTOR, p-EIF4EBP1, p-RPS6KB1, p-RPS6, TPX2, and DAZL were significantly decreased in oocytes of *Sphk1/2*^*gc−/−*^ mice after hCG stimulation (Fig. 6f–h). Interestingly, the addition of EGF and S1P significantly increased the protein levels of p-Akt, p-mTOR, p-EIF4EBP1, p-RPS6KB1, p-RPS6, TPX2, and DAZL in oocytes from cultured COCs (Fig. 6f–h). S1P, but not EGF, also increased these protein levels in oocytes from cultured DOs (Fig. 6f–h). The inhibition of S1PR1/3 by VPC23019 had no effect on S1P-induced Akt activation in oocytes (Fig. S11). Next, we assessed the effect of S1P treatment during in vitro maturation (IVM) on embryonic development after in vitro fertilization (IVF). Both the two-cell rate and the blastocyst rate were significantly increased when wild-type COCs were matured in the presence of S1P (Fig. 6i). The two-cell rate was also significantly increased when *Sphk1/2*^*gc−/−*^ COCs were matured in the presence of S1P (Fig. 6j). These results suggest that SphK-produced S1P in somatic cells activates the Akt/mTOR cascade reaction in oocytes and promotes TPX2 translation and oocyte developmental competence.

## Discussion

Our findings demonstrate that SphK-produced S1P, in response to LH-EGFR signaling, elevates intracellular calcium levels in somatic cells to induce oocyte meiotic maturation. Somatic cell-derived S1P also improves oocyte developmental competence, possibly by the Akt/mTOR-mediated maternal mRNA translation.

Oocytes within preovulatory follicles maintain meiotic arrest by NPR2-produced cGMP in response to NPPC [1]. LH triggers EGF-like growth factors to activate EGFR [7] and then decreases NPR2 activity and cGMP levels in granulosa cells, resulting in meiotic resumption. In our study, LH-EGFR signaling-activated SphK1/2 in granulosa cells to increase S1P levels, consistent with a previous study showing that EGF enhances S1P synthesis and secretion in glioblastoma stem cells [17]. The inhibition of SphK1/2 by SKI-II blocked the effects of LH-EGFR signaling on S1P production and oocyte meiotic resumption. Furthermore, the depletion of *Sphk1/2* in granulosa cells decreased the levels of S1P and the rate of oocytes with meiotic resumption. Thus, LH-EGFR signaling induces oocyte meiotic resumption by SphK-produced S1P. S1P could induce oocyte meiotic resumption by elevating the intracellular calcium levels of cumulus cells to decrease the binding affinity of NPR2 for NPPC [9, 10]. The elevation of intracellular calcium by S1P might also cause NPR2 inactivity by dephosphorylation [6, 29]. The depletion of *Sphk1/2* in granulosa cells could not block the meiotic resumption of all oocytes within preovulatory follicles, possibly because the LH/hCG-induced decrease in NPPC levels in MGCs leads to a reduction in NPR2 function [30].

S1P, a physiological calcium elevator, can mobilize Ca^2+^ from the ER by S1P receptors and non-receptor pathways [18]. In our study, S1P could elevate intracellular calcium levels by increasing PLN levels to inhibit SERCA2 activity. The low levels of S1P in *Sphk1/2*^*gc−/−*^ ovaries may cause the decrease of calcium levels in cumulus cells by the reduced PLN. PLN levels could be regulated by its mRNA transcription [31] and by its protein degradation induced by E3 ubiquitin-protein ligase 1 [32]. However, the increase in S1P by hCG treatment had no effect on the mRNA levels of *Pln*, indicating that S1P increases PLN levels in cumulus cells by preventing its protein degradation. S1P may stabilize PLN protein by inhibiting its ubiquitination [33]. Treatment with hCG also increased the phosphorylation levels of SphK1/2 and the protein levels of PLN in MGCs. This will cause the initial cGMP decrease in MGCs by elevating calcium levels to inactivate NPR2 [6]. PLN knockout mice are fertile [34], indicating that S1P could also elevate calcium levels of cumulus cells by other signaling(s). Although the inhibition of highly expressed S1PR1 and S1PR2 could not block S1P-elevated calcium levels, we could not exclude the roles of other three receptors (S1PR3-5) in S1P-elevated calcium levels of cumulus cells. In addition, the PLN homologous proteins SLN and MLN may also involve in S1P-elevated calcium levels of cumulus cells (Fig. S12).

The LH-EGFR signaling-activated Akt/mTOR pathway enhances spindle-related mRNA translation in oocytes [13], which is involved in promoting oocyte developmental competence [12]. The mTOR signaling pathway is significantly downregulated in the oocytes of patients with polycystic ovary syndrome (PCOS) [35]. Our in vivo and in vitro data demonstrated that LH-EGFR signaling-produced S1P in somatic cells activated the Akt/mTOR cascade reaction in oocytes, increased the protein levels of TPX2, and promoted oocyte developmental competence. The blockade of S1P production by *Sphk1/2* depletion in granulosa cells caused the reduced levels of p-Akt, p-mTOR, and TPX2, a defect in spindle assembly of MII oocytes, and the failure of early embryo development. It is possible that low levels of S1P in somatic cells of *Sphk1/2*^*gc−/−*^ mice decreased Akt/mTOR activity in oocytes, resulting in abnormal spindle assembly and then infertility [13, 36, 37]. Thus, LH-EGFR signaling activates SphK to produce S1P in somatic cells, and then S1P promotes oocyte developmental competence, possibly by the activation of the Akt/mTOR pathway in oocytes. S1PR signaling can activate the Akt/mTOR pathway to enhance mRNA translation in different cell types [20, 21]. S1PR3 expression was the highest in oocytes, and the inhibition of S1PR1/3 by VPC23019 could not block S1P-induced Akt activation in oocytes. This may be because the five S1P receptors have redundant, overlapping, or compensatory functions [38].

**Fig. 7:**
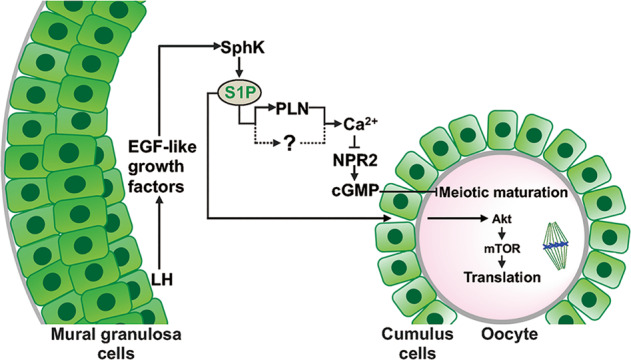
Model of the signaling pathways involved in somatic cells control of oocyte meiotic maturation and developmental competence. In preovulatory follicles, LH stimulates the expression of EGF-like growth factors in mural granulosa cells to activate EGFR in cumulus cells. EGFR signaling causes SphK activation to produce S1P. S1P elevates the calcium levels of cumulus cells via PLN, S1P receptors, and/or undefined signaling(s) to decrease NPR2 activity and cGMP levels, resulting in oocyte meiotic maturation. In addition, S1P secreted from cumulus cells activates the Akt/mTOR cascade reaction in oocytes, and promotes oocyte spindle-related mRNA translation and developmental competence.

In conclusion, LH-EGFR signaling activates SphK to produce S1P in somatic cells, which is critical for oocyte meiotic maturation and developmental competence via different pathways (Fig. 7). Our findings reveal the mechanism by which S1P promotes oocyte maturation and provide a molecular basis for the potential clinical application of S1P in improving oocyte quality.

## Materials and methods

### Animals and chemicals

WT C57/BL6 mice were purchased from the Guangdong Medical Laboratory Animal Center (Guangzhou, China). *Sphk1*^*−/−*^ mice (stock number 019095) and *Sphk2*^*−/−*^ mice (stock number 019140) were purchased from the Jackson Laboratory (Bar Harbor, ME, USA). To generate granulosa cell-specific deletion of *Sphk2* mice, exons 4 and 6 of *Sphk2* were flanked with LoxP sites (Fig. S5b), and *Sphk2* ^*fl/fl*^ mice were crossed with *Fshr-Cre* mice [39]. Finally, *Sphk2*^*fl/fl*^*; Fshr-Cre* mice were crossed into the *Sphk1*^*−/−*^ background, yielding *Sphk1*^*−/−*^; *Sphk2*^*fl/fl*^*; Fshr-Cre* (*Sphk1/2*^*gc−/−*^) mice (Fig. S5c). The primers used for genotyping are shown in Table S3. Female mice (21–23 days old) were injected with 5 IU equine chorionic gonadotropin (eCG) 48 h before use to stimulate follicle development. In some experiments, female mice were treated with 5 IU eCG, followed by 5 IU human chorionic gonadotropin (hCG) 48 h later to induce ovulation. All animal protocols were approved by the Institutional Animal Care and Use Committee of the South China University of Technology. All reagents were purchased from Sigma-Aldrich (St. Louis, MO, USA) unless otherwise stated.

### Isolation and culture of follicles, COCs, and denuded oocytes

Large antral follicles (350–400 µm) isolated from eCG-primed mice using a 29**-**gauge needle were cultured on Millicell inserts (Millipore, Billerica, MA, USA) supplemented with LH (1 µg/mL), AG1478 (1 µM), and/or SKI-II (5–20 µM). COCs isolated from eCG-primed mice were cultured in 24-well plates supplemented with NPPC (30 nM), EGF (10 ng/mL), SKI-II, AG1478, S1P (20 µM), W146 (1 µM), and/or JTE-013 (5 µM). Denuded oocytes (DOs) were separated from the COCs by repeatedly drawing the oocytes in and out of a glass pipette slightly smaller in diameter than the oocyte and then cultured in 50 µL drops of M16 medium (M7292) supplemented with EGF, S1P, and/or VPC023019 (5 µM). The follicle and COC culture medium was bicarbonate-buffered MEMα with Earle salts (Thermo Fisher Scientific, Waltham, MA, USA) supplemented with 100 IU/ml penicillin–streptomycin, 0.23 mM pyruvate, and 3 mg/ml bovine serum albumin (BSA). For follicle culture, 1% ITS (I3146) was added. All cultures were carried out at 37 °C in an atmosphere of 5% CO_2_. At the indicated time points, cumulus cells of COCs and DOs were collected for gene and protein analyses. In some experiments, COCs were collected at 2 h of culture to analyze the levels of calcium, S1P, cGMP, and the binding affinity of NPR2 for NPPC. Oocyte meiotic resumption was assessed at 4 h of culture by scoring the released oocytes for GVB after the removal of cumulus cells. To observe the morphology of cumulus expansion, the follicles were cultured in a medium for 8 h and the COCs were released from the follicles, and the COCs were cultured in a medium with 5% fetal bovine serum (FBS, Thermo Fisher Scientific) instead of a BSA for 15 h. Images were obtained using a Zeiss 7000 microscope (Carl Zeiss, Oberkochen, Germany).

### Superovulation, fertilization, and fertility assays

For superovulation analysis, the oocyte-cumulus masses were harvested from the oviducts of WT and *Sphk1/2*^*gc−/−*^ female mice at 13 h post-hCG, and the oocytes were categorized and counted. For in vitro fertilization (IVF), COCs isolated from eCG-primed mice were cultured in a medium supplemented with or without S1P for 0.5 h and then cultured in an S1P-free medium for an additional 15.5 h. DOs obtained by treating COCs with 3 μg/ml hyaluronidase were fertilized with normal sperm isolated from fertile adult male mice in an HTF medium. Then, the fertilized oocytes were cultured in KSOM (MR-121-D) medium. The number of two-cell stage embryos was scored 24 h after IVF, and the number of blastocyst stage embryos was scored on Days 4–5 of the culture. In some experiments, zygotes collected from WT and *Sphk1/2*^*gc−/−*^ females were cultured in KSOM medium to continuously observe their embryonic developmental potential. Blastocysts were stained with DAPI to quantify the number of total cells. All cultures were carried out at 37 °C in an atmosphere of 5% CO_2_. Images of embryos at different stages were obtained using a Zeiss 7000 (Carl Zeiss). For the fertility test, 8-week-old WT or *Sphk1/2*^*gc−/−*^ female mice were housed with 8-week-old fertile male mice for up to 8 months. The number of pups per litter was recorded at birth, and the average cumulative number of pups per female was calculated at the end of the fertility test.

### RNA isolation and analysis

Total RNA was isolated and purified from MGCs, cumulus cells, and oocytes using the RNeasy micro-RNA isolation kit (Qiagen, Valencia, CA, USA) according to the manufacturer’s instructions. Reverse transcription was performed directly after RNA isolation using the QuantiTek reverse transcription system (Qiagen). qRT–PCR was conducted to quantify the steady-state mRNA levels using a Light Cycler 96 instrument (Roche, Basel, Switzerland). Target gene expression was calculated based on the 2^−ΔΔCt^ method using ribosomal protein L19 (*Rpl19*) and/or glyceraldehyde-3-phosphate dehydrogenase (*Gapdh*) as the endogenous control. For RNA-seq analysis, MGCs and cumulus cells were collected from WT and *Sphk1*/2^*gc−/−*^ mice at 2 h post-hCG. RNA was extracted from these samples, and was analyzed by Annoroad Gene Technology Co., Ltd. (Beijing, China). The qRT–PCR primers are shown in Table S3.

### Western blotting

Total proteins of MGCs were extracted in WIP buffer (Cell Chip Biotechnology, Beijing, China), and 15–25 μg protein was used for sample loading. Cumulus cells from 50–150 COCs or 50–300 oocytes were lysed in an SDS loading buffer. These protein samples were separated by SDS–PAGE and transferred to a PVDF membrane (Millipore). The membranes were blocked in TBST (Tris-buffered saline with Tween 20) buffer containing 5% milk, and then were incubated with primary antibodies (Table S4) overnight at 4 °C. After rinsing with TBST buffer, the membranes were incubated with appropriate secondary antibodies (1:5000, Zhongshan Golden Bridge Biotechnology, Beijing, China). Finally, the bands were detected using the SuperSignal West Pico Kit (Thermo Fisher Scientific) and visualized by the Tanon 5200 chemiluminescent imaging system (Tanon, Shanghai, China). The relative intensity of the bands was quantified by ImageJ software (NIH Image, Bethesda, MD, USA). GAPDH or α-tubulin (Tubulin) was used as an internal control. Uncropped scans of representative blots are shown in supplementary original blots.

### Immunofluorescence and ovarian histology assays

Ovaries isolated from eCG-primed mice or eCG-primed mice followed by hCG were fixed in 4% paraformaldehyde (PFA), embedded in paraffin, and sectioned at 5 μm. The sections were dewaxed, rehydrated, and subjected to antigen retrieval using 0.01 M sodium citrate buffer (pH 6.0). After blocking in 10% normal donkey serum, the sections were incubated with primary antibodies (Table S4) followed by Alexa Fluor 488-conjugated secondary antibodies (1:200, Thermo Fisher Scientific). Finally, the sections were counterstained with DAPI. To visualize microtubules and chromosomes, oocytes were fixed in 4% PFA and permeabilized with PBS containing 0.5% Triton X-100 for 30 min. After blocking with 10% normal donkey serum for 1 h at room temperature, oocytes were incubated with Alexa Fluor 488-conjugated anti-α-tubulin antibodies (1:200, Abcam) and counterstained with DAPI. Digital images were captured using a Zeiss LSM 800 confocal microscope (Carl Zeiss), and the fluorescence intensity was analyzed using Zeiss Zen 3.0 software. The exposure time was kept the same for the control and mutant samples. The fluorescence intensity of MGCs and cumulus cells was calculated by averaging the fluorescence signals of MGCs and cumulus cells in each large antral follicle. The relative fluorescence intensity was analyzed after background subtraction.

For histological analysis, paraffin-embedded ovaries were serially sectioned at 5 μm and stained with periodic acid/Schiff (PAS) reagent and haematoxylin. The numbers of large antral follicles, corpus luteum, and oocytes having undergone GVB were counted by examining serial sections through the entire ovary.

### Measurement of S1P levels

Ovaries and COCs were isolated from eCG-primed mice or mice at 2 h post-hCG. In some experiments, COCs from eCG-primed mice were collected after different drug treatments for 2 h. More than 1 mg/mL ovarian protein samples or 1500 COC samples per group were used for analyzing S1P levels according to previous reports [19, 40]. Briefly, 12.5 µL of sample suspension, 5 µL of internal standards (1 ng/mL for C17-S1P), and 32.5 µL of methanol were added to a 1.5 mL tube. The mixture was centrifuged at 14,000 × *g* for 15 min, then 10 µL of the supernatant was injected into the liquid chromatography–tandem mass spectrometry system (LC-MS/MS; LC, Shimazu Nexera X2 LC-30AD, Shimadzu, Japan; MS, AB SCIEX QTRAP 4500, AB SCIEX, MA, USA) equipped with an electrospray ionization (ESI) source and multiple reaction monitoring (MRM) for analysis.

### Measurement of the intracellular calcium levels

The intracellular calcium levels were monitored by using Fluo 3-AM (Dojindo Laboratories, Kumamoto, Japan), as reported before [9, 10]. Briefly, COCs with different treatments were collected and then incubated with 5 µM Fluo 3-AM for 20 min. Samples were examined by a Nikon A1 confocal microscope (Nikon, Tokyo, Japan) or a Zeiss LSM 800 confocal microscope (Carl Zeiss). All quantifications were performed using Nikon NIS Elements BR 5.10 software or Zeiss Zen 3.0 software. The fluorescence intensity (pseudocolor) in cumulus cells was analyzed after background subtraction, which represents the intracellular calcium levels.

### Measurement of the binding affinity of NPR2 for NPPC

The binding affinity of NPR2 for NPPC was monitored by using mono-5-[and 6]-carboxyfluorescein-labeled NPPC (FAM-NPPC; Phoenix Pharmaceuticals, Belmont, CA) as reported before [9]. Briefly, COCs with different treatments were collected and incubated with 100 nM FAM-NPPC for 30 min, and then the fluorescence-labeled ligand binding was assessed by a confocal microscope (Nikon A1 or Zeiss LSM 800). The fluorescence intensity (green) in cumulus cells was analyzed after background subtraction, which represents the binding affinity of NPR2 for NPPC.

### Measurement of cGMP levels in COCs

COCs from eCG-primed mice were collected after different drug treatments for 2 h. 100 COC samples per group were solubilized in 200 µL HCl (0.1 M) on ice for 10 min, and then were thawed and centrifuged at 12,000 × *g* for 5 min. The supernatant was collected to a tube and dried in an oven at 60 °C. The levels of cGMP were determined using a cGMP enzyme immunoassay kit obtained from Cayman Chemicals (Ann Arbor, MI, USA).

### P4 level assay

The blood from WT and *Sphk1/2*^*gc−/−*^ mice at 48 h post-hCG was collected by cardiac puncture, and the serum progesterone levels were analyzed by Beijing North Biotechnology Institute (Beijing, China).

### Statistical analysis

All experiments were performed at least three times independently, and the data were represented as means ± s.d. GraphPad Prism software (v8.4.0, La Jolla, CA, USA) were used to perform the statistical analysis and graph generation. The statistical significance was analyzed either by unpaired two-tailed Student’s *t*-test (two-group comparison) or by one-way ANOVA followed by Tukey’s Honestly Significant Difference (more than two groups). Values were considered significantly different if *P* < 0.05.

## Supplementary information

Supplemental Materials

Supplementary Figure 1

Supplementary Figure 2

Supplementary Figure 3

Supplementary Figure 4

Supplementary Figure 5

Supplementary Figure 6

Supplementary Figure 7

Supplementary Figure 8

Supplementary Figure 9

Supplementary Figure 10

Supplementary Figure 11

Supplementary Figure 12

Supplementary Table 1

Supplementary Table 2

Supplementary Table 3

Supplementary Table 4

Original Blots

## Data Availability

RNA-seq data have been submitted to the NCBI Gene Expression Omnibus (GEO) under accession number GSE215989. All data supporting the findings of this study are available within the article and/or the Supplementary Materials. Additional data related to this paper may be requested from the authors.
