# Supplementary material for: SphK-produced S1P in somatic cells is indispensable for LH-EGFR signaling-induced mouse oocyte maturation
Source: Cell Death Dis. 2022 Nov 17;13(11):963. doi: 10.1038/s41419-022-05415-2 (PMC9671891; doi:10.1038/s41419-022-05415-2)
Supplement: Supplementary file 1 — Supplemental Materials [file 41419_2022_5415_MOESM1_ESM.docx]

**Supplemental Materials**

**SphK-produced S1P in somatic cells is indispensable for LH-EGFR signaling-induced mouse oocyte maturation**

Feifei Yuan, et al.

**Supplementary Figure Legends**

**Fig. S1 The effect of SKI-II on LH- and EGF-induced oocyte meiotic resumption and cumulus expansion-related gene expression.** **a,** Specific detection of SphK1, SphK2, p-SphK1 and p-SphK2 antibodies. The ovaries were isolated from *Sphk1^-/-^; Sphk2^fl/fl^* mice for SphK1/2 and p-SphK1/2 (green) staining. The nuclei were counterstained with DAPI (blue). **b,** Proportion of oocytes having undergone GVB at 4 h of culture. Large antral follicles from eCG-primed mice were cultured in medium supplemented with 1 μg/mL LH and different doses of SKI-II. COCs from eCG-primed mice were cultured in medium with the presence of 30 nM NPPC, supplemented with 10 ng/mL EGF and different doses of SKI-II. **c, d,** Quantitative RT-PCR analysis for cumulus expansion-related gene expression at 4 h of follicle culture (**c**) and at 6 h of COC culture (**d**). At end of the follicle culture, the COCs were released from the follicles. *Rpl19* and *Gapdh* were used as internal references for each biological repeat. **e,** Proportion of oocytes having undergone GVB at 4 h of culture. COCs from eCG-primed mice were cultured in medium with the presence of NPPC, supplemented with EGF, 0.5 mM tetracaine, 10 µM 2-APB, 20 µg/mL heparin and/or 20 μM SKI-II. For **a–e**, n = 3 independent experiments. The data represent the mean ± s.d. *P* values were determined by one-way ANOVA followed by Tukey’s test (**b–e**). ns, not significant. Scale bars, 100 μm.

**Fig. S2 S1P reverses the inhibition of SKI-II on EGF functions.** COCs isolated from eCG-primed mice were cultured in medium, supplemented with NPPC, EGF, SKI-II, and/or 20 μM S1P. **a,** Proportion of oocytes having undergone GVB was determined at 4 h of culture. (n = 3 independent experiments). **b–e,** Representative images of calcium levels (pseudocolor, **d**) and NPR2 affinity for NPPC (green, **e**) in cumulus cells at 2 h of culture, and the statistical analysis of calcium levels (**b**) and NPR2 affinity (**c**). (n ≥ 15 independent samples). The data represent the mean ± s.d. *P* values were determined by one-way ANOVA followed by Tukey’s test (**a–c**). ns, not significant. Scale bars, 100 μm.

**Fig. S3 The effects of hCG and S1P on the expression of PLN in granulosa cells.** **a,** Quantitative RT-PCR analysis for relative concentrations of *Pln*, *Sln* and *Mln* in granulosa cells isolated from eCG-primed mice. **b,** Quantitative RT-PCR analysis for *Pln* levels in MGCs and cumulus cells (CC) isolated from the mice before (no hCG group) and after hCG treatment (2 h). **c,** Western blotting analysis of PLN levels in MGCs and cumulus cells isolated from hCG-treated mice. GAPDH was used as an internal control. **d,** Western blotting analysis of PLN levels in cumulus cells from cultured COCs with and without S1P. GAPDH was used as an internal control. For **a–d**, n = 3 independent experiments. The data represent the mean ± s.d. *P* values were determined by two-sided Student’s *t*-test (**b,** **d**) and one-way ANOVA followed by Tukey’s test (**c**). ns, not significant.

**Fig. S4 The effects of S1PR inhibitors on EGF- and S1P-increased PLN and calcium levels in cumulus cells.** **a,** Quantitative RT-PCR analysis for relative concentrations of *S1pr1-5* in MGCs, cumulus cells (CC), and oocytes (OO) isolated from eCG-primed mice. (n = 3 independent experiments). **b,** Western blotting analysis of PLN levels in cumulus cells from cultured COCs. COCs isolated from eCG-primed mice were cultured in medium with the presence of NPPC, supplemented with EGF, 1 µM W146 and/or 5 µM JTE-013 for 2 h. GAPDH was used as an internal control. (n = 3 independent experiments). **c–d,** Representative images (**c**) and statistical analysis (**d**) of calcium levels (pseudocolor) in cumulus cells at 2 h of culture. (n ≥ 16 independent samples). The data represent the mean ± s.d. *P* values were determined by one-way ANOVA followed by Tukey’s test (**b,** **d**). ns, not significant. Scale bars, 100 μm.

**Fig. S5 The knockout strategy and efficiency of *Sphk2*-conditional knockout mice.**

**a,** The effect of EGF on meiotic resumption of oocytes collected from *Sphk1^-/-^* and *Sphk2^-/-^* mice at 48 h post-eCG. Proportion of oocytes having undergone GVB was determined at 4 h of culture. (n = 3 independent experiments). **b,** A schematic diagram illustrating the loxP site insertion for the generation of *Sphk2^fl/fl^* mice. **c,** DNA graph represented PCR results of different alleles. **d, e,** Detection of SphK2 knockout efficiency in granulosa cells from eCG-primed mice using immunofluorescence (**d**) and western blotting (**e**). Granulosa cells were collected for western blotting analysis. (n = 3 independent experiments). GAPDH was used as an internal control. The data represent the mean ± s.d. *P* values were determined by two-sided Student’s *t*-test. Scale bars: 100 μm.

**Fig. S6 The effects of *Sphk1/2* deletion on follicular development and ovulation.** **a, b,** Ovarian histology (**a**) and quantification of the number of large antral follicles (**b**) from eCG-primed mice. (n = 5 independent experiments). **c, d,** Ovarian histology (**c**) and quantification of the number of corpora lutea (CLs, **d**) from 8-month-old WT and *Sphk1/2^gc-/-^* mice. Representative images are shown. (n = 5 independent experiments). **e,** Quantitative RT-PCR analysis for cumulus expansion-related gene expression in WT and *Sphk1/2^gc-/-^* mice at 4 h post-hCG. (n = 3 independent experiments). **f,** Serum progesterone (P4) concentrations in WT and *Sphk1/2^gc-/-^* mice before and after hCG injection. (n = 3 independent experiments). **g,** Quantitative RT-PCR analysis for *Pln* mRNA levels in MGCs and cumulus cells (CC) isolated from WT and *Sphk1/2^gc-/-^* mice at 2 h post-hCG. (n = 3 independent experiments). The data represent the mean ± s.d. *P* values were determined by two-sided Student’s *t*-test (**b, d, e–g**). ns, not significant. Scale bars: 100 μm.

**Fig. S7 The effects of *Sphk1/2* deletion on the levels of calcium, the affinity of NPR2 and the expression of NPPC and NPR2. a–c,** Representative images of calcium levels (pseudocolor) and NPR2 affinity for NPPC (green) in cumulus cells from WT and *Sphk1/2^gc-/-^* mice at 2 h post-hCG (**a**), and the statistical analysis of calcium levels (**b**) and NPR2 affinity (**c**). (n ≥ 18 independent samples). Scale bars: 100 μm. **d–e,** The mRNA (**d**) and protein (**e, f**) levels of NPPC and NPR2 in MGCs (**d** and **e**) and cumulus cells (CC, **d** and **f**) isolated from WT and *Sphk1/2^gc-/-^* mice at 4 h post-hCG (n = 3 independent experiments). The data represent the mean ± s.d. *P* values were determined by two-sided Student’s *t*-test (**b–f**). ns, not significant.

**Fig. S8 The analysis of transcriptome integrity in WT and *Sphk1/2^gc-/-^* mice.** **a, b,** Bubble chart illustrating the enriched KEGG associated with the significantly downregulated transcripts in *Sphk1/2^gc-/-^* MGCs (**a**) and cumulus cells (**b**) identified by RNA-seq. **c,** Gene set enrichment analysis (GSEA) revealing the enrichment of terms in *Sphk1/2^gc-/-^* cumulus cells relative to WT. NES, normalized enrichment score. **d-f,** GSEA revealing the enrichment of cell cycle (**d**), transcriptional regulation (**e**) and oxidative phosphorylation (**f**) in *Sphk1/2^gc-/-^* cumulus cells relative to WT. **g, h,** Bar graph illustrating the enriched GO/KEGG terms associated with the significantly upregulated transcripts in *Sphk1/2^gc-/-^* MGCs (**g**) and cumulus cells (**h**) identified by RNA-seq. Transcripts with a fold-change ≥ 2 and a *P* value ≤ 0.05 were selected for analysis.

**Fig. S9 The staining of MII oocytes and blastocysts in WT and *Sphk1/2^gc-/-^* mice.** **a, b,** Immunofluorescent staining of α-tubulin showing ovulated oocytes with defective cytokinesis (**a**) or remaining in telophase I (**b**). The enlarged views of the white boxed area indicate morphology of microtubules (green) and chromosomes (blue). Representative images are shown. **c, d,** Representative images of DAPI (blue) staining of blastocysts (**c**) and quantification of the total cells in the blastocysts (**d**). (n = 5 independent experiments). The data represent the mean ± s.d. *P* values were determined by two-sided Student’s *t*-test. Scale bars, 20 μm.

**Fig. S10 The in vitro maturation defect of oocytes in *Sphk1/2^gc-/-^* mice.** COCs isolated from eCG-primed mice were cultured in medium supplemented with NPPC and EGF for 16 h. **a,** Immunofluorescent staining of α-tubulin showing spindle assembly in MII oocytes after in vitro maturation (IVM). The enlarged views of the white boxed area indicate microtubules (green) and chromosomes (blue). Representative images are shown. Scale bars, 20 μm. **b,** Quantification of the rate of MI and MII oocytes and the rate of normal spindle formation in MII oocytes after IVM. The numbers of analyzed oocytes are indicated (n). The data represent the mean ± s.d. *P* values were determined by two-sided Student’s *t*-test.

**Fig. S11** **Western blotting analysis of Akt phosphorylation levels in oocytes from different treatments.** Denuded oocytes were collected from eCG-primed mice and then were cultured in medium supplemented with S1P and/or 5 µM VPC23019 for 1.5 h. Total Akt were used as an internal control. The data represent the mean ± s.d. *P* values were determined by one-way ANOVA followed by Tukey’s test.

**Fig. S12 The effect of *Pln* deletion on the mRNA levels of *Sln* and *Mln* in granulosa cells.** *Pln* knockout mice (*Pln^-/-^*) were purchased from Cyagen Biosciences Co., Ltd. (Suzhou, China). **a,** The mRNA levels of *Pln* were not detected in the granulosa cells of *Pln^-/-^* mice. (n = 3 independent experiments). **b,** The mRNA levels of *Sln* and *Mln* were significantly increased in the granulosa cells of *Pln^-/-^* mice. (n = 3 independent experiments). The data represent the mean ± s.d. *P* values were determined by two-sided Student’s *t*-test.
