# Supplementary material for: SphK-produced S1P in somatic cells is indispensable for LH-EGFR signaling-induced mouse oocyte maturation
Source: Cell Death Dis. 2022 Nov 17;13(11):963. doi: 10.1038/s41419-022-05415-2 (PMC9671891; doi:10.1038/s41419-022-05415-2)

Supplemental Material – original blots

SphK-produced S1P in somatic cells is indispensable for LH-EGFR signaling-induced mouse oocyte maturation

Feifei Yuan, et al.

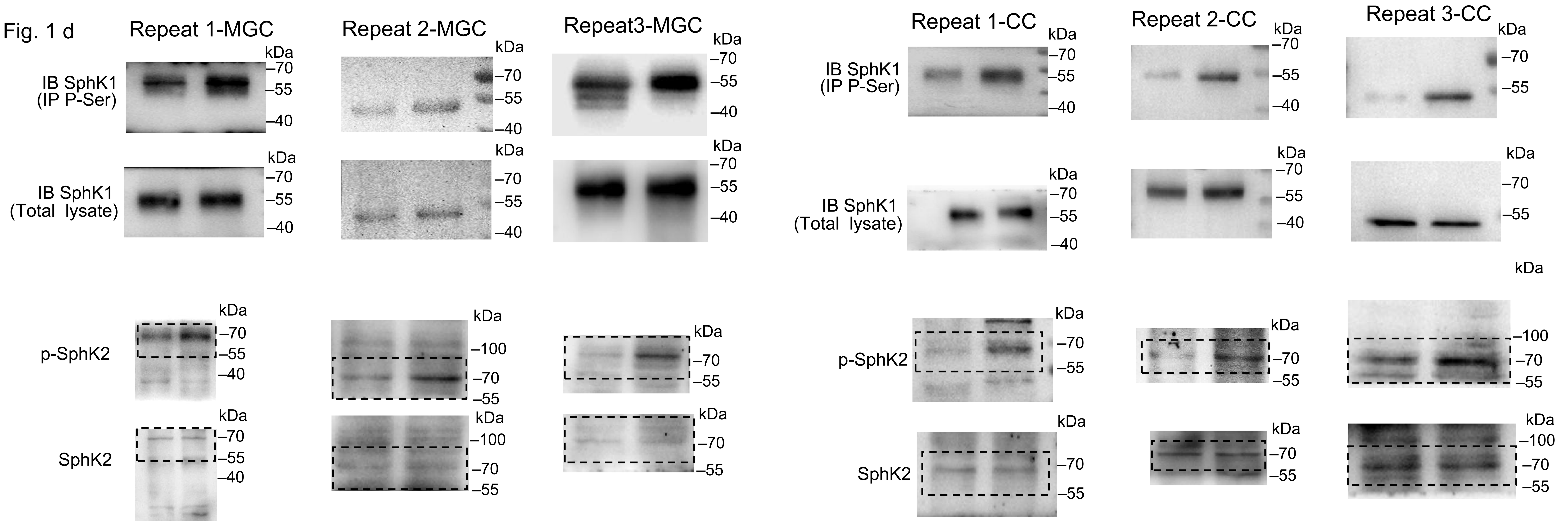

Repeat 1 is a representative image of Fig. 1 d.

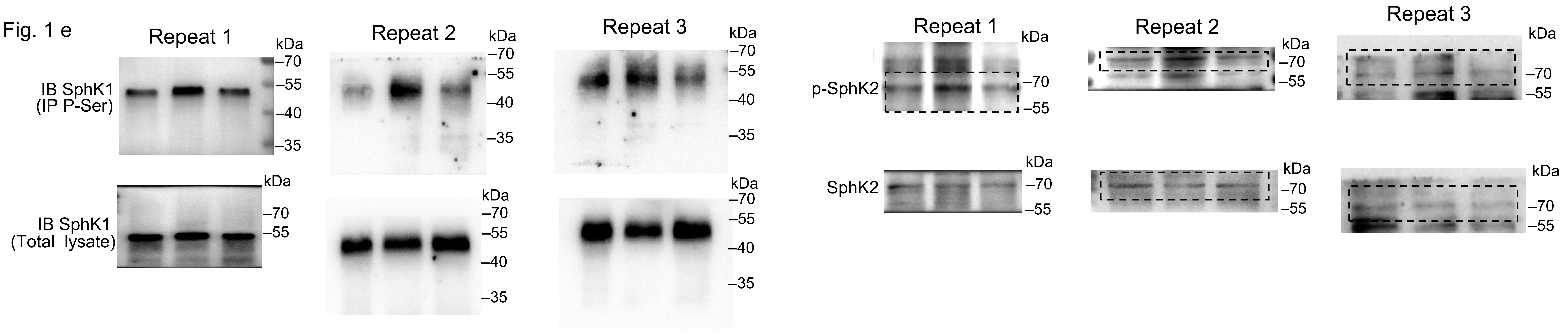

Repeat 1 is a representative image of Fig. 1 e.

Fig. 1 f

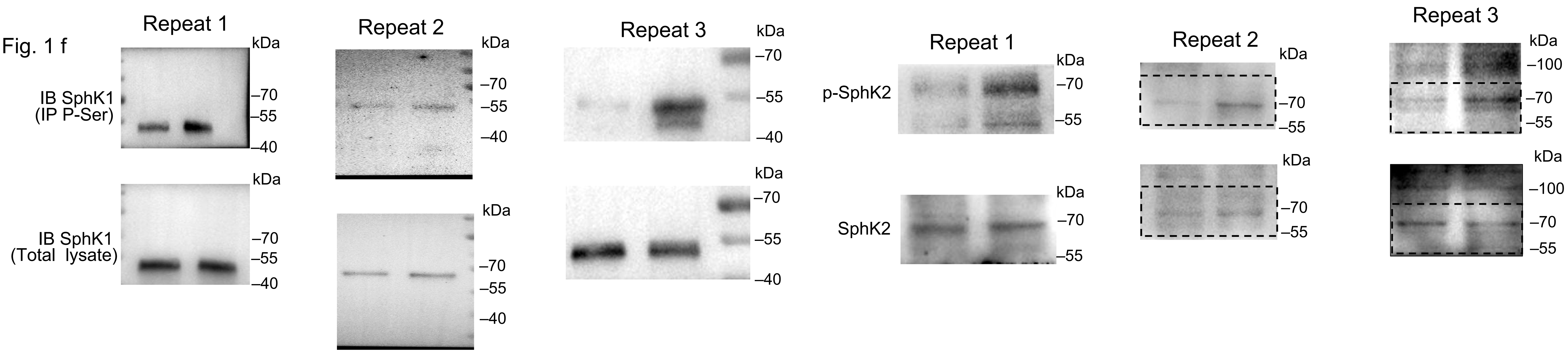

Repeat 1 is a representative image of Fig. 1 f.

Fig. 3 d

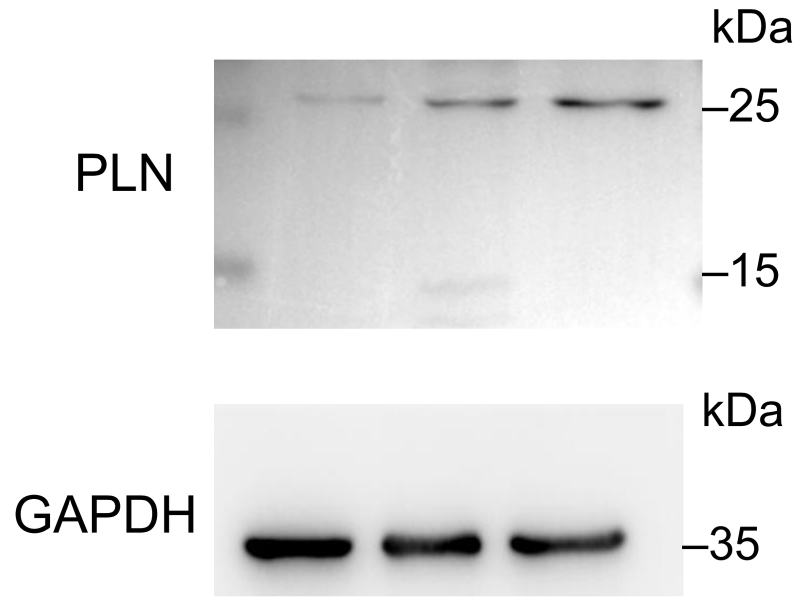

Fig. 3f

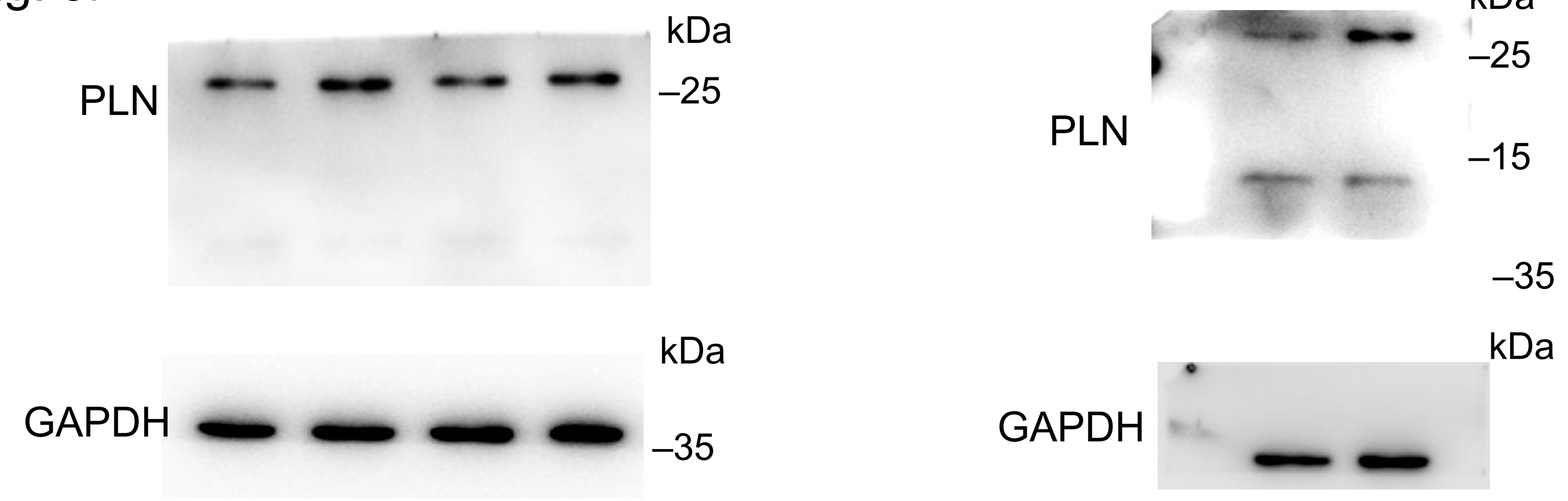

Fig. 4 e

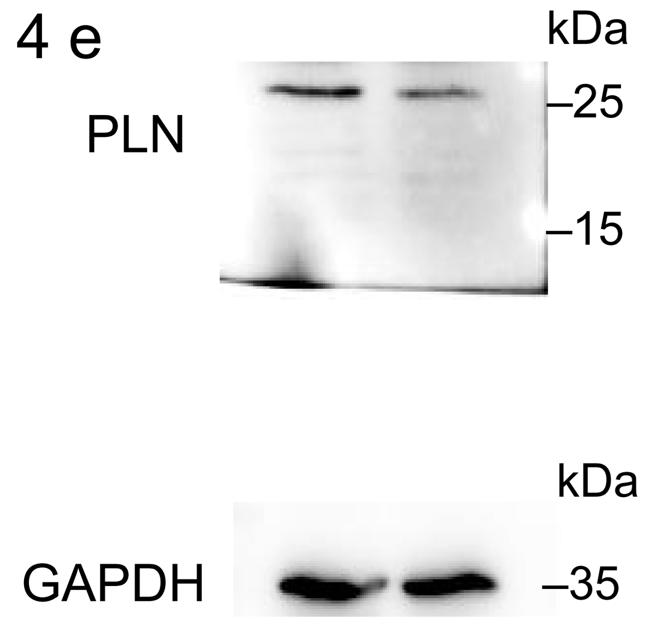

Fig. 3 e

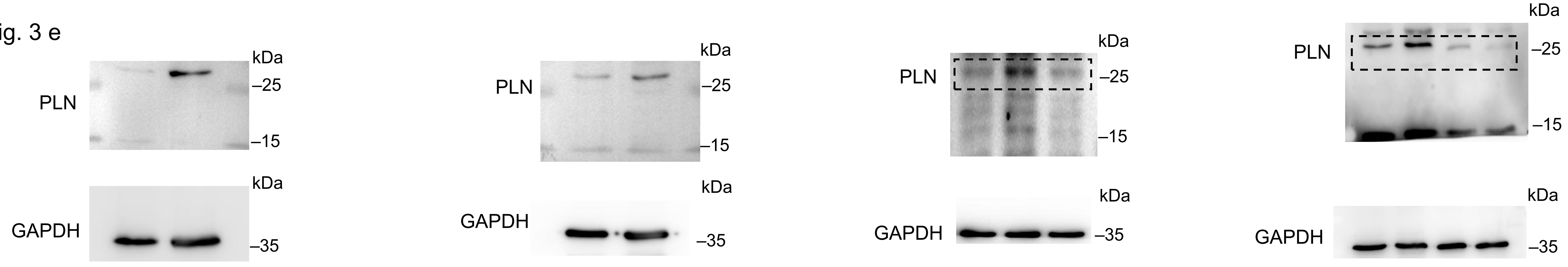

Fig. 6 f

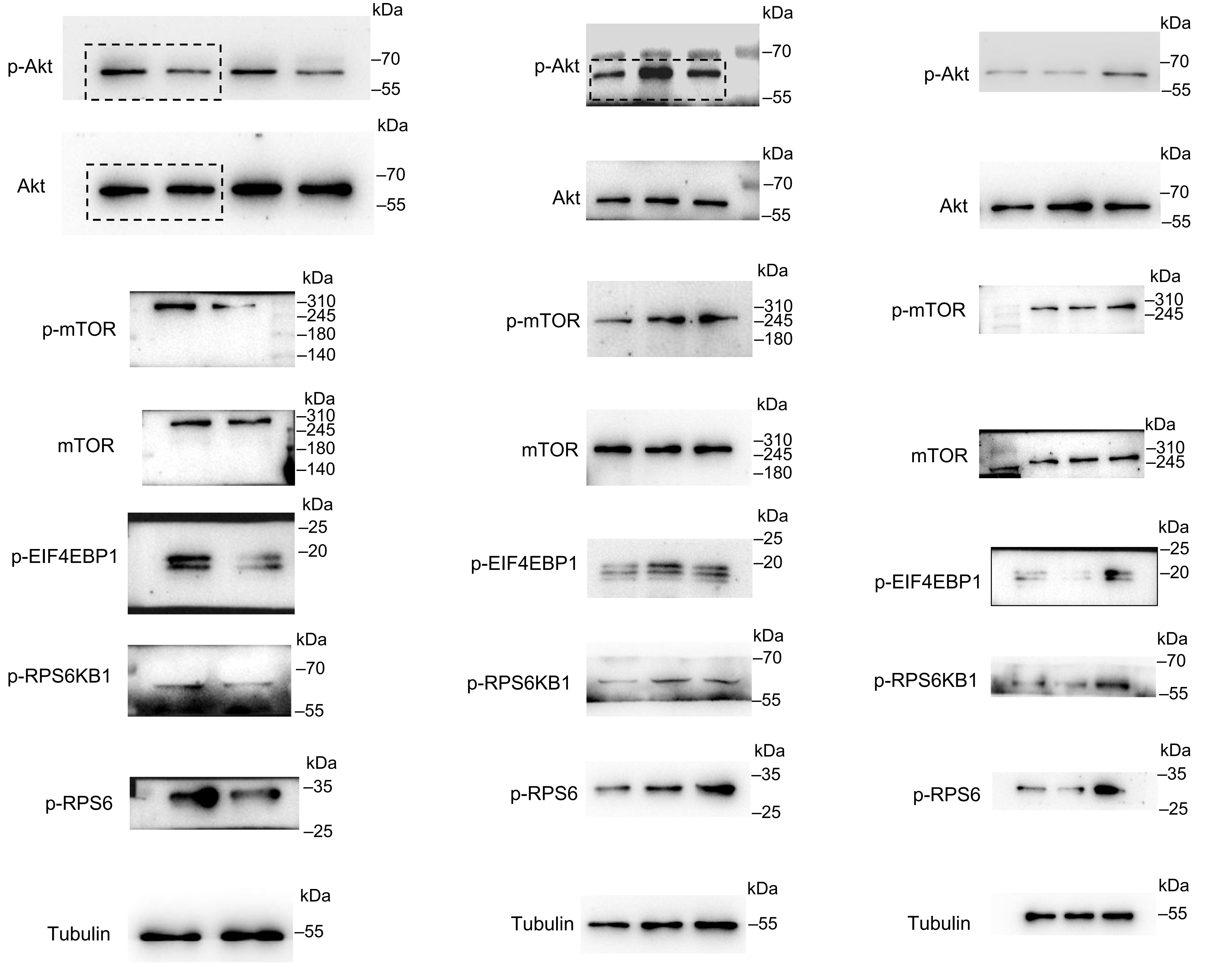
p-Akt
Akt
p-mTOR
mTOR
p-EIF4EBP1
p-RPS6KB1
p-RPS6
Tubulin
kDa
70
55
310
245
180
140
25
20
35

Fig. 6 h

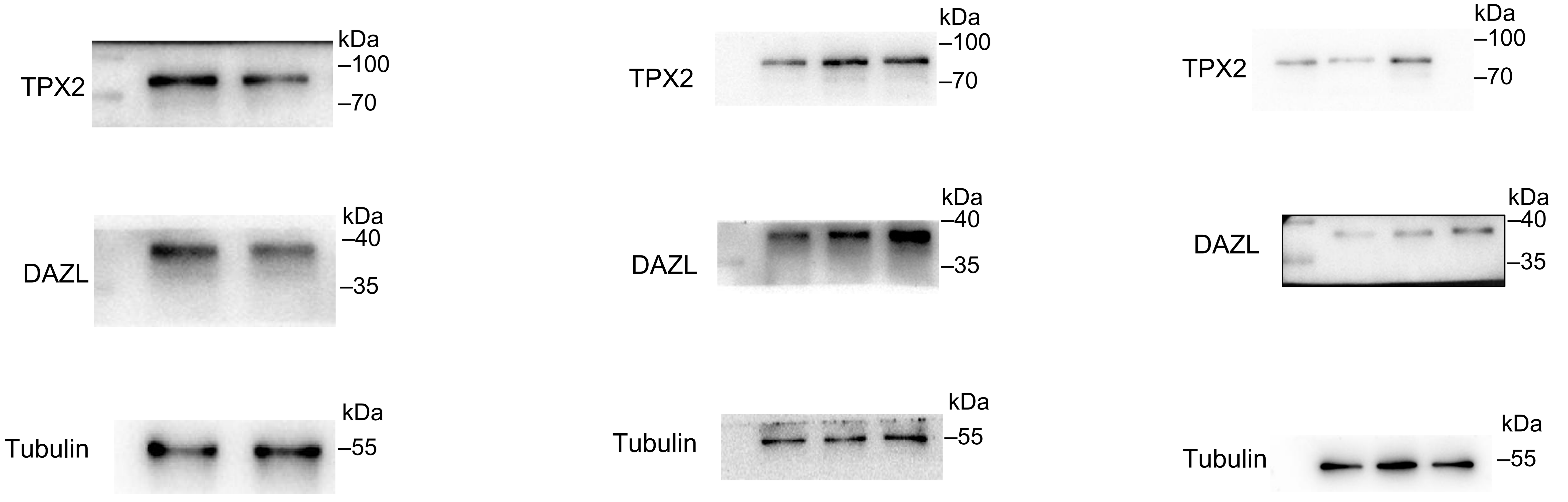
TPX2
DAZL
Tubulin
kDa
100
70
40
35
55

Fig. S3 c

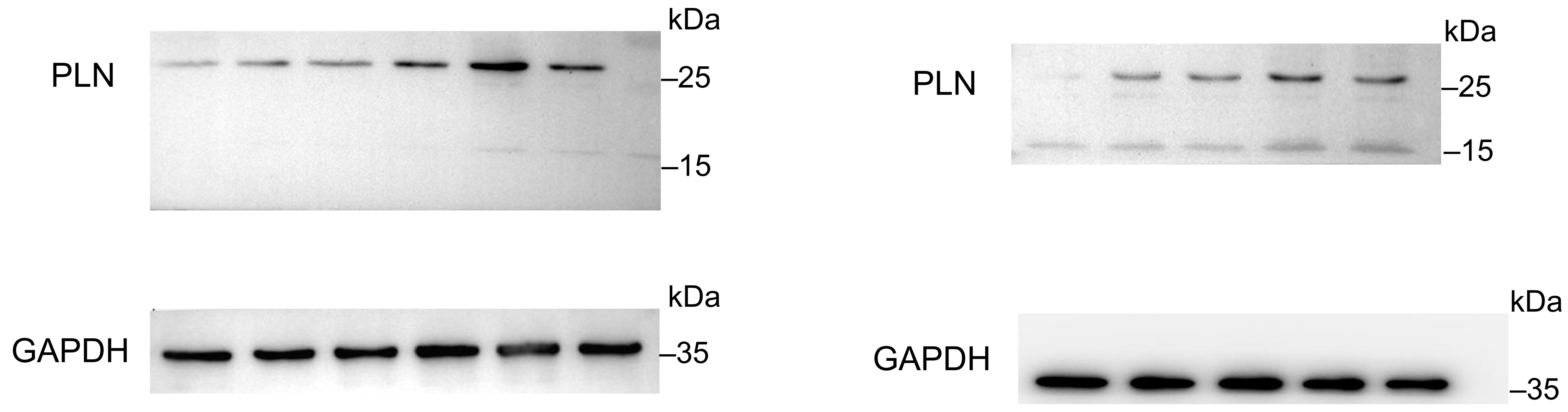

Fig. S3 d

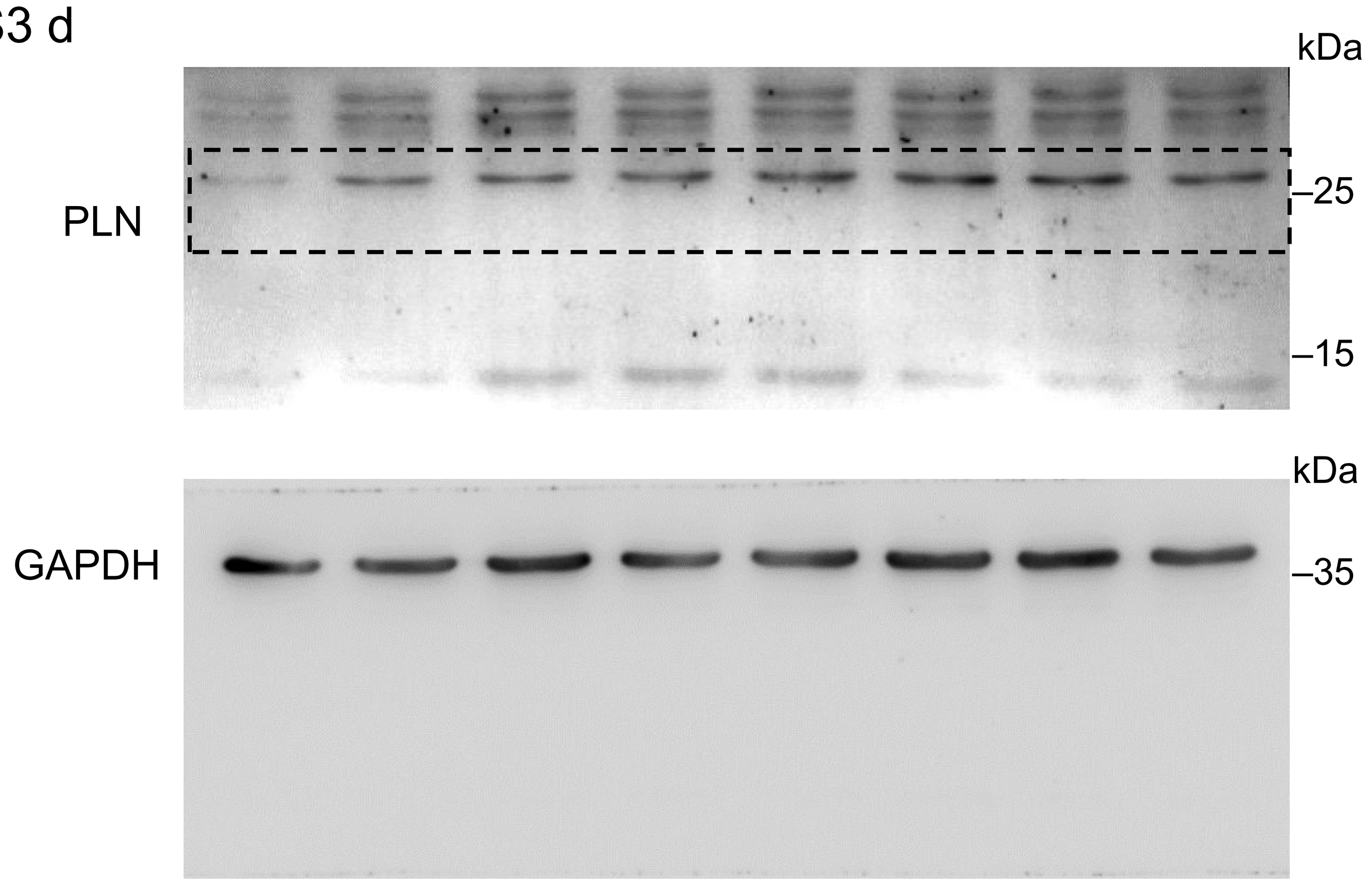

Fig. S4 b

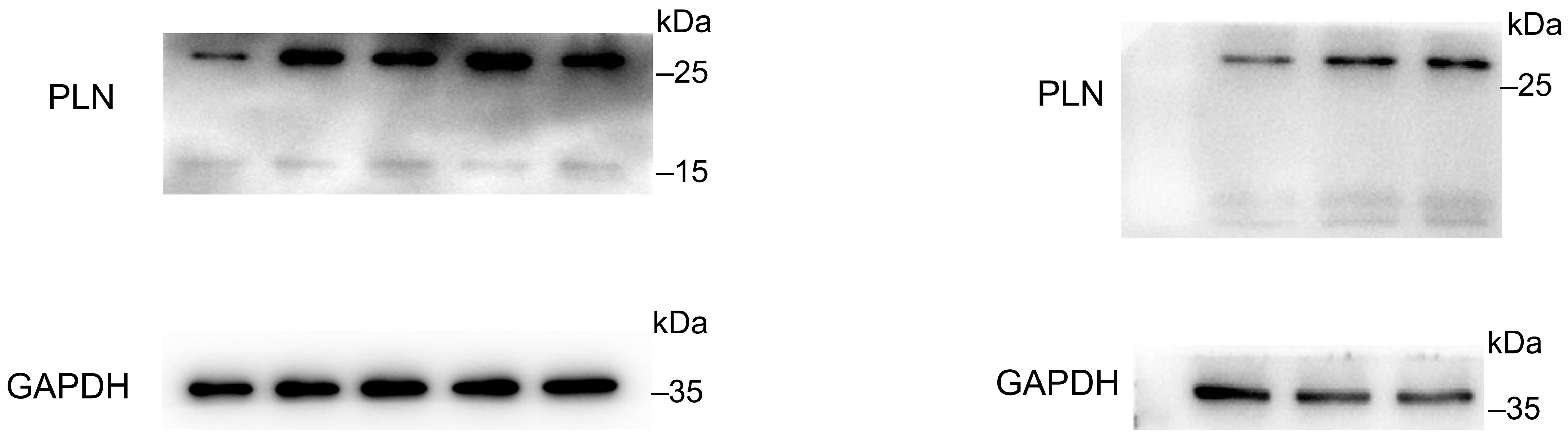

Fig. S5 e

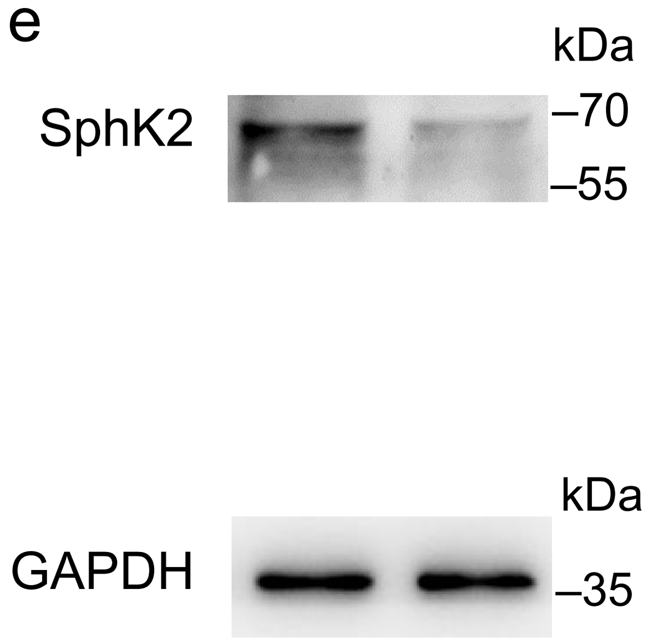

Fig. S7 e

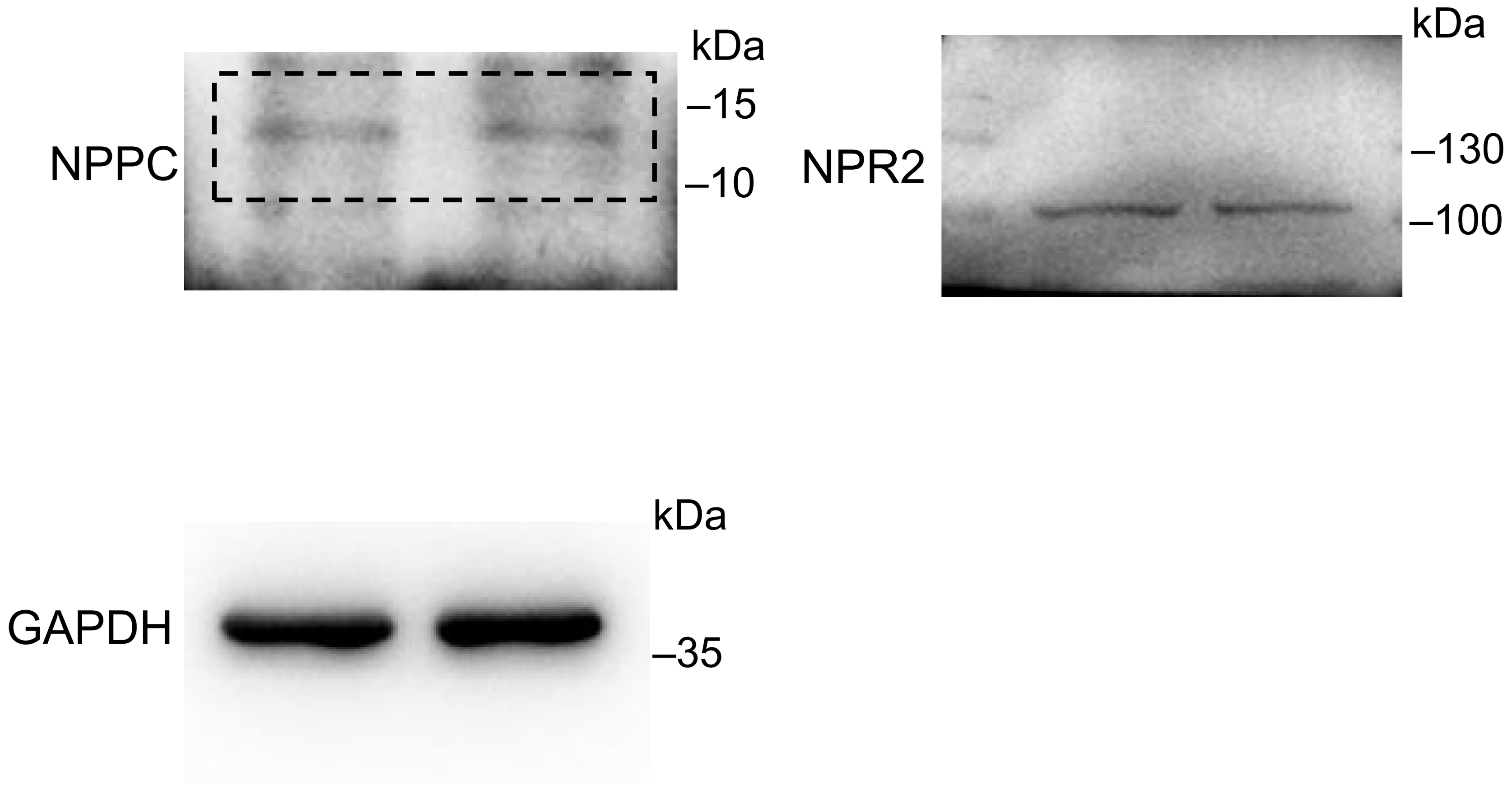

Fig. S7 f

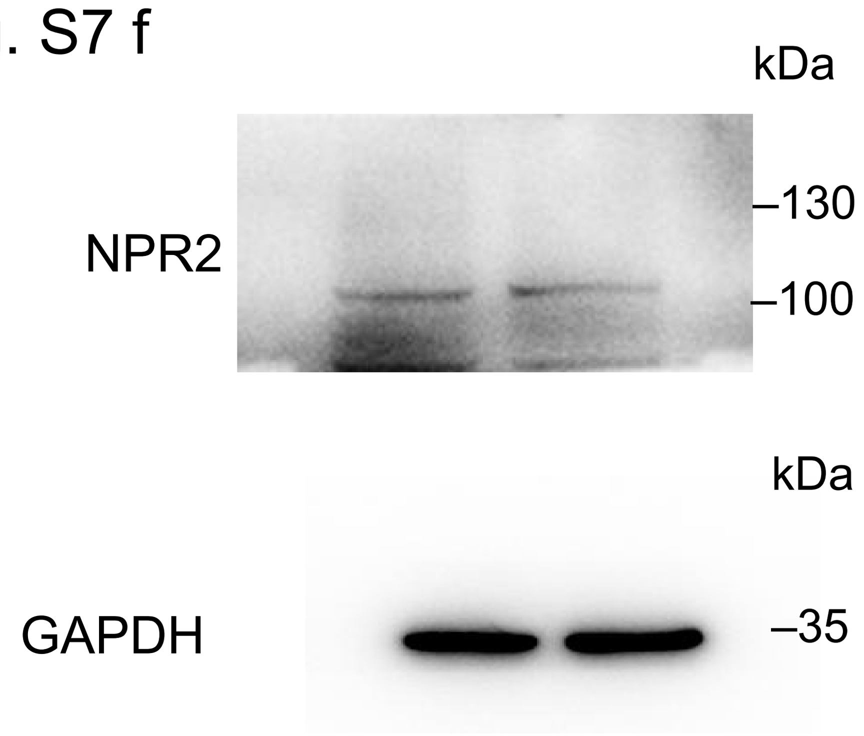

Fig. S 11

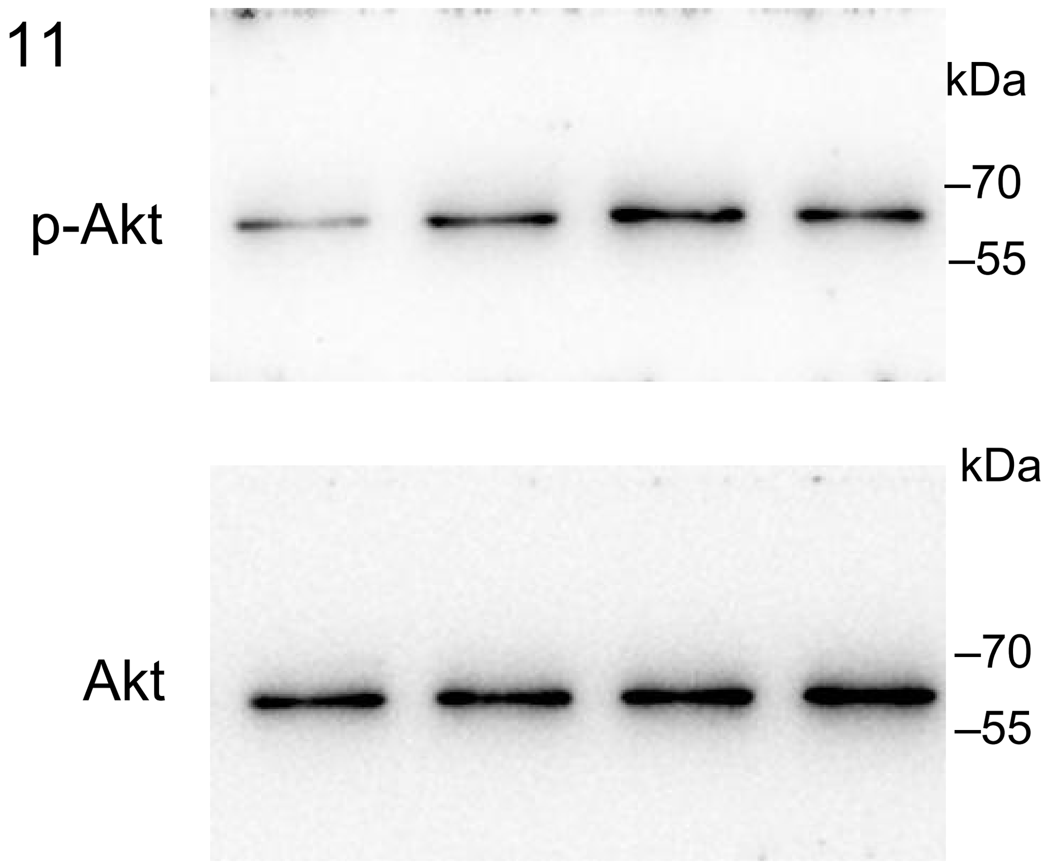

Supplement: Supplementary file 18 — Original Blots [file 41419_2022_5415_MOESM18_ESM.pdf]
